# Supplementary material for: Beyond checklists: Using clinic ethnography to assess the enabling environment for tuberculosis infection prevention control in South Africa
Source: PLOS Glob Public Health. 2022 Nov 9;2(11):e0000964. doi: 10.1371/journal.pgph.0000964 (PMC10022266; doi:10.1371/journal.pgph.0000964)
Supplement: S1 Checklist — (DOCX) [file pgph.0000964.s001.docx]

Inclusivity in global research

PLOS’ policy on inclusivity in global research aims to improve transparency in the reporting of research performed outside of researchers’ own country or community and ensures that PLOS publications reporting global research adhere to high standards for research ethics and authorship. Authors of relevant research articles may be asked to complete the questionnaire below, which outlines ethical, cultural, and scientific considerations specific to inclusivity in global research. This questionnaire may be requested when researchers have travelled to a different country to conduct research, if research uses samples collected in another country, research with Indigenous populations or their lands, or if research is on cultural artefacts. Researchers travelling to another country solely to use laboratory equipment will not normally be required to complete the questionnaire. However, the questionnaire can be requested at the journal’s discretion for any submission – if you have been requested to complete this questionnaire by the PLOS journal you submitted to, please do so.

Please complete the questionnaire below and include this as a Supporting Information file with your manuscript. Note that if your paper is accepted for publication, this checklist will be published with your article in the supporting information files. Please ensure that you reference the checklist in the main body of your manuscript. We suggest adding a subsection ‘Inclusivity in global research’ to your Methods section and adding the following sentence: “Additional information regarding the ethical, cultural, and scientific considerations specific to inclusivity in global research is included in the Supporting Information (SX Checklist)”

The questions have been designed to be applicable to a wide range of study types, and there are subsections for both human subjects research and non-human subjects research. If any of the questions are not relevant to your research please mark them as “N/A” as appropriate.

**Ethical considerations, permits and authorship**

*This section is applicable to all research types.*

Provide details as to who granted permissions and/or consent for the study to take place in the Methods section of your manuscript. This should include the names of **all** ethics boards, governmental organizations, community leaders or other bodies that provided approval for the study. If individuals provided approval refer to these people by their role or title but do not list their name(s).

Reported on page number: 12-13, Lines 250 - 268

If there were any deviations from the study protocol after approval was obtained please provide details of these changes in the Methods section of your manuscript.
Did this study involve local collaborators that are residents of the country where the research was conducted or members of the community studied? If you do not have any authors from said communities, please provide an explanation for this below.

N/A

Yes, the broader project (Umoya Omuhle, described on page 6, lines 134-138) was a collaboration between UK and South African institutions. The South African partners were from the University of Cape Town and the University of KwaZulu-Natal. The qualitative and ethnographic work conducted in the context of this project is one of 7 work packages. Dr Hayley MacGregor and Dr Anna Voce are South African nationals, and Dr Voce was the South African lead for the qualitative data collection and is listed as a co-author on the paper. We acknowledge the inputs of the two South African field assistants who contributed to data collection, but did not contribute to this particular manuscript, in terms of meeting the PLOS criteria for authorship.

Everyone listed as an author should meet PLOS’ criteria for authorship and all individuals who meet these criteria should be included in the author byline, rather than the acknowledgements. Authorship criteria is based on the International Committee of Medical Journal Editors (ICMJE) Uniform Requirements for Manuscripts Submitted to Biomedical Journals - for further information please see here: <https://journals.plos.org/plosone/s/authorship>.

**Human subjects research (e.g. health research, medical research, cross-cultural psychology)**

Did you obtain written informed consent from a representative of the local community or region before the research took place? How did you establish who speaks for the community? Details of written informed consent obtained from study participants should be reported separately in the Methods section of your manuscript.

Written gatekeeper permission to conduct the study was provided at the provincial level and from each of the three health districts within which 6 clinics were located. Informed written consent for clinic participation in the study was sought from the clinic manager of each clinic, prior to the start of fieldwork/data collection.

How did members of the local community provide input on the aims of the research investigation, its methodology, and its anticipated outcome(s)?

The broader project (Umoya Omuhle) was a 3-year collaboration between UK and South African partners. We held meetings prior to submitting the proposal to discuss research scope, questions, roles of partners and expectations. Post-submission, we held an inception meeting and subsequent meetings on a yearly basis in Cape Town and Durban to develop research protocols, train local researchers, discuss emerging findings and the analysis plan, and draft outlines for outputs for the project. We met virtually on a monthly basis to track progress on the project activities as well as outputs.

When engaging with the local community, how did you ensure that the informed consent documents and other materials could be understood by local stakeholders?

KK to address

Will the findings of the research be made available in an understandable format to stakeholders in the community where the study was conducted (e.g. via a presentation, summary report, copies of publications, etc.)? Please provide details of how this will be achieved.

As reported on lines 257-264: Informed written consent for clinic participation in the study was sought from the clinic manager of each clinic, prior to the start of fieldwork/data collection. On entry to each clinic, researchers held an information session with all staff prior to the start of data 259 collection. This was usually incorporated into the regular staff meeting, to inform clinic staff of all researcher activities while on site. All questions pertaining to the study purpose and processes were addressed in the information session, and as they arose during the fieldwork. Members of staff engaged in informal conversations could thus be informed regarding the nature of the research and their participation to ensure transparency regarding the data collection generated through the ethnographic method.

The key findings of this study, alongside the results of the wider project, were collated and lay summaries fed back to participants via presentations delivered locally. Please see the project summary brief used in the feedback sessions to the clinics in Western Cape and KwaZulu-Natal here: <https://www.lshtm.ac.uk/research/centres-projects-groups/uo#resources>

All academic publications arising from the research project were published fully open access ensuring immediate access to findings online.

**Non-human subjects research using specimens/ animals collected as part of the study, or those housed in archival collections. Examples include archaeology, paleontology, botany and zoology.**

Did the permission you obtained from a local authority to perform the study include an agreement on access to outputs and benefit sharing? This may include procedures to enable fair distribution of the benefits and resources arising from the research performed. Please include any details of Prior Informed Consent and Benefit Sharing Agreements obtained. These may be required by field-specific regulations, for example the Convention on Biological Diversity (CBD) and the associated Nagoya Protocol.

N/A

If the material used in your study was imported, please A) provide the year it was imported and B) indicate whether permits were obtained to import/export the materials used, C) provide details of any permits obtained. If this information is not available, please indicate this.

N/A

If you used archival specimens, please state how the material used in your study was acquired by the institute it is held in and provide details of any permits obtained for the original excavations/ sample collection. If this information is not available, please indicate this.

N/A

How was the potential cultural significance of the materials collected in your study to local communities considered in your research design? Were Indigenous peoples and/or local researchers and institutions involved with archaeological excavations / collection of specimens? If so, please provide a description of their involvement.

N/A

If your manuscript includes photographs of human remains please indicate whether authors obtained permission from descendants or affiliated cultural communities to do so.

N/A
