# Supplementary material for: Beyond checklists: Using clinic ethnography to assess the enabling environment for tuberculosis infection prevention control in South Africa
Source: PLOS Glob Public Health. 2022 Nov 9;2(11):e0000964. doi: 10.1371/journal.pgph.0000964 (PMC10022266; doi:10.1371/journal.pgph.0000964)
Supplement: Attachment — Submitted filename: Beyond Checklists_Response to reviewers_020822.docx [file pgph.0000964.s002.docx]

­­­­Dr Stella Arakelyan

School of Health Sciences

University of Dundee

Dundee, Scotland

Prof. Karina Kielmann

Institute of Tropical Medicine

Antwerp,

Belgium

­­­

2.08.2022

**Subject: Revision and resubmission of the manuscript: PGPH-D-22-00745**

Dear Editor,

Thank you for the invitation to revise and resubmit our manuscript “Beyond checklists: using clinic ethnography to assess the enabling environment for tuberculosis infection prevention control in South Africa”. We are grateful to the reviewers for their feedback and insightful comments. We have addressed all the comments raised and believe this has strengthened our manuscript.

We hope the revised manuscript is now considered suitable for publication in PLOS Global Public Health.

Sincerely

Dr Stella Arakelyan

Prof. Karina Kielmann

| **Responses to Reviewers' Comments** | |
| --- | --- |
| **Reviewer 1 comment** | **Author response (please note all page/line numbers refer to the ‘clean copy’ of the revised manuscript)** |
| Methods  Page 8, Table 2:  1.It would be helpful to the reader to include a brief description of how rural, semi-rural, peri-urban, and urban were defined in the main text for the purposes of clinic selection. | A footnote has been added at the bottom of Table 2 that reads:  *The clinic location is based on the classification adopted by the National Treasury Republic of South Africa in Local Government Budgets and Expenditure Review (2011). Based on this classification, rural refers to sparsely populated areas in which people depend on farming, natural resources and migratory labour, remittances and social grants; semi-rural refers to areas that are functionally rural and have traditional tenure systems; urban refers to large towns with an urban core; peri-urban refers to areas on the periphery of an urban area, with both formal and informal housing.* |
| Page 8, Table 2:  It is not clear how the governance structures of "KZN government" and "KZN Provincial government" clinics differ. Were only provincial clinics selected due to convenience? | The governance structure for all clinics should read the same – KZN government. All clinics selected in KZN fall under provincial authority. Clinics under local authority are atypical in KwaZulu-Natal. |
| Page 8, Table 2:  The observation periods are quite short for ethnography. Typically, ethnographic observation extends over a period of weeks to reduce the Hawthorne effect, yet, the data do not suggest being observed resulted in a change in behavioural patterns. It would be worth adding a few sentences under methods about how the observation was carried out to provide readers with a bit more context. | Thank you for this pertinent comment. Indeed, we had limited time in the clinics, however, our observations were relatively structured in terms of noting pertinent features of clinic spaces, processes, interactions and practices that had bearing on IPC. This enabled researchers to capture the observations in fieldnotes during the time spent in clinics, which in turn formed the basis of the thick descriptions. We have added a few sentences in lines 187-197, page 9 to reflect this explanation, as follows:  *Using a guide that was loosely structured around the parameters of recommended TB-IPC measures, we conducted observations in each of the six facilities, in particular focusing on waiting rooms, reception areas and clinical consultation rooms. Observations in these spaces explored (a) the material differences in spatial layout; (b) workplace design and ventilation; (c) staff and patient movements around spaces and measures taken to encourage or discourage patient congregation (e.g., triaging, fast-tracking); (d) power dynamics and the interactions between staff members, patients and staff members and amongst patients. Although our time in the facilities was limited, the relatively structured focus in observations allowed the researchers to capture pertinent features of clinic spaces, processes, interactions and practices that had bearing on TB-IPC through fieldnotes.* |
| Page 9, line 183: I found it a bit confusing when the authors referred to the researchers as “participating” in the fieldwork, as “participants” are often those who are the subject of the research. Consider replacing with “conducted” or “undertook.” | We have replaced ‘participated’ with ‘conducted’ in line 178, page 9. |
| Page 9, line 202: what is meant by “service users”? Is this strictly patients, or might healthcare workers be considered service users in this context? | By ‘service users’, we meant individuals accessing the primary care clinics included in our research. To avoid confusion, we have now changed this to ‘patients’ in line 207, page 9. |
| Page 9, 2nd paragraph: - Did interviews happen before or after observation? - How were informal conversations documented? | We have added clarification on these questions in lines 188-190, page 9, as follows:  *During their time in the clinics, researchers observed their surroundings and took notes on clinic features and processes as pertinent to the implementation of TB IPC. Interviews were conducted with relevant health care staff when possible and convenient.*  And further, in lines 209-211, page 10:  *Researchers captured the essence of these conversations through fieldnotes. These notes were integrated with the notes from our observations and formed the basis of ‘thick descriptions’.* |
| Ethical considerations  Lines 246-263: It would be helpful to include a few lines about how the use of data from informal conversations would be covered under institutional approvals given the nature of ethnographic work. | A sentence has been added starting at lines 262-264, page 12 to expand upon the point regarding the efforts made to ensure awareness of the nature of the research and the broader range of data collection as part of the ethnographic method. The right to withdrew or not participate in all of the research elements is covered in the last sentence of the ethics section. The sentence reads as:  *Members of staff engaged in informal conversations could thus be informed regarding the nature of the research and their participation to ensure transparency regarding* *the data generated through the ethnographic method.* |
| Results  Line 324: “In other clinic…” should read “In another clinic…” | We have changed ‘other clinic’ to ‘another clinic’ in line 330, page 15. |
| Lines 368-371: These two sentences were a bit confusing to read. Consider re-wording to make the point a bit clearer. | Thanks for pointing this out to us. We have modified this in lines 372-378, page 17 as follows:  *Similarly, the effectiveness of designated IPC champions was not always evident. For example, one of the IPC champions we met was unaware of the checklist of TB-IPC measures they were tasked with monitoring. In Clinic 3, the IPC manager mentioned that TB-IPC guidelines were an integral part of the general IPC file, but that these had last been reviewed in 2012. While this manager was aware that routine monitoring of TB-IPC measures was meant to occur every six months, the most recent TB-IPC checklist had been completed­­­­­ 18 months prior to our visit.* |
| Line 399: What is meant by staff working “at secondary level”? Does this mean outside primary care? | We have revised this sentence in lines 405-408, page 18:  *Conversely, the manager in Clinic 6 reported that they had initiated regular TB screening of staff. This measure was intended to improve on past practice when only new staff, or those working in secondary hospitals and not primary care, were screened for TB.* |
| Line 412: I was unclear on what the authors meant by “registering nurses seeing two streams for patient care at the same time”. Does this mean one nurse had two sets of patients that they would move between, or two nurses each seeing patients at the same time in the same room? | We meant that nurses were seeing two streams of patients in the same room at the same time. The line 421, page 19 now reads as:  *In Clinic 2, the registering nurses were seeing two streams of patients at the same time in the same room due to staff shortages.* |
| Line 482: “patient TB test results” should read “TB patient test results.” | We have made a minor change in line 493-494, page 22 to clarify that we are referring to the speed of reporting of test results in individuals as yet undiagnosed:  *In Clinic 3, one doctor described how he was lobbying to have a TB testing laboratory and equipment to speed up the reporting of individuals’ test results.* |
| Lines 543-545: It may flow better to stop the sentence leading into the quotation on lines 547-48 at “Another staff member had misperceptions about types of face-coverings:” and then to add the rest of the sentence after the quotation with some additional context, e.g. “The same staff member also reported disposing N95s after each use as soon as practically possible, suggesting that…” | We have made suggested changes in lines 555-563, page 24:  *The same staff member also reported disposing N95 masks after each use as soon as practically possible, suggesting a potential lack of awareness on proper usage, disposal and reuse of respiratory protective devices.* |
| Discussion  Given the nature of the enquiry, the authors may want to consider saying a little more about a few TB IPC “recommended practices” that may not be ideal or may require more nuance for the setting, e.g.: - Line2 424-426 includes data on introducing appointment dates rather than times. The concept of patient appointments is often recommended by the privileged without the understanding that a vast majority of public sector patients rely on public transport (ie an informal taxi network), which does not run according to a schedule, making appointment times near to impossible to keep, especially for those living outside of busy transport hubs. | Thanks for this comment and suggestion. The ‘recommended practices’ you refer to relate to insights provided by the local clinic managers who shared the sentiment that the introduction of appointment systems might help to (1) optimise service provision, and (2) address overcrowding/patient congregations increasing the risk of TB transmission. There was indeed awareness of the challenges, in particular relating to transport. We have added 2 sentences to highlight that in lines 441-445, page 20; those read as:  *There was also awareness from service providers that patients relying on public sector clinics are likely to be reliant on public transport, which in many instances is limited and does not adhere to reliable schedules. This can make it challenging to stick to rigid appointment times and requires services to adapt to contextual realities.* |
| Line 496 refers to health workers preferring personal comfort, in this case using air conditioning units to keep out heat. This can also be extended to keeping windows open or asking sick patients to wait in outside queues during winter months, which may also bring a level of discomfort and lack of feeling safe for patients or staff that is also overlooked by those who advise on IPC. Might the global community need to rethink some of these easy to say IPC practices, but not so easy to endure for those who are on the receiving end? | Thank you for this important comment regarding local applicability and acceptability of ‘global solutions’. We try to emphasise in our paper that the optimal implementation of IPC multimodal strategies requires good understanding of local constraints and conditions. We have added a clause to emphasise this point in the conclusion section in lines 708-711, page 30 as follows:  *There is a pressing need to adapt the implementation of multimodal measures recommended by WHO (2019) to local structural and operational constraints of primary health clinics, as well as considerations of security and comfort for staff and individuals attending clinics.* |
| Limitations  It would be helpful to remind readers that the data were collected pre-COVID, and thus practices may have improved as a result of the COVID-19 pandemic; nonetheless the lessons remain important for sustaining and improving IPC impetus. | We have added this point to our limitations. Please refer to lines 699-703, pages 29-30 to read changes:  *Finally, our fieldwork was undertaken before the global outbreak of COVID-19, and some IPC practices in clinics, for example, mask-wearing, are likely to have changed as a result of new directives. Nonetheless, our insights on the wider dynamics of systems hardware and software remain important for sustaining and improving TB-IPC practices in resource-constrained TB-burdened settings.* |
| It would be worth considering how presenting the purpose of the work to all clinic staff (as indicated under methods) might have affected (or not) the observed behaviour. May the timing of presentation and observation of mitigated this? – or perhaps it was a testament to how normalized it may be not to follow IPC protocols if behaviour remained unchanged despite the purpose of the observation being known. | The presentation of the purpose of the work occurred on entry to each clinic. In one clinic, attempts to adopt IPC protocols, specifically mask wearing, were observed immediately after the information session, but were not sustained throughout the period of the field visit. These observations were indeed recorded in the thick case descriptions. The comment of the reviewer is apt – Non-adherence to IPC protocols was indeed observed as the usual or default behaviour. |
| **Reviewer 2 comments** |  |
| As is sometimes the case with qualitative/ethnographic findings, the thickness of descriptions could get in the way of finding key information quickly. I would suggest a table or an illustrative diagram to summarize the results, with a representative quote where possible. | In our paper, the description of findings, with inclusion with representative quotes to illustrate the point we are making, follows the conventional standards of reporting qualitative (ethnographic) research. We see your point, however, and have added an illustrative diagram (Fig. 1) to highlight specifically the hardware/software interactions across the hierarchy of controls for IPC. |
| An additional level of synthesis of results could also be helpful. One suggestion would be to merge the managerial and administrative control findings together. | Thank you for the suggestion. We have chosen not to integrate the managerial and administrative control measures as we feel they are quite different – the former relating to governance aspects while the latter more associated with service delivery procedures and processes. We have added this point to explain why we kept these two types of controls distinct on page 13, lines 274-277:  *Although managerial and administrative controls are sometimes combined, we have reported on these separately as they relate to different aspects of organising TB care and TB-IPC (governance versus service delivery).* |
